# The Public Health

**Published:** 1918-08-24

**Authors:** 


### THE PUBLIC HEALTH.

### Bubonic Plague.

The recent arrival of a ship at the Port of London from Calcutta with four cases of bubonic plague emphasises the necessity of constant supervision with regard to ships coming from infected areas, and the need for adequate precautionary measures in this country. The finding of dead rats on board the infected ship indicates the method of infection and the manner in which the home rat may become infected with the specific organism of plague. The rat serves no useful purpose in modern civilised life. Prior to modern improvements in sanitation it no doubt fulfilled the purpose of a useful scavenger, and wherever there is laxity in removing and destroying refuse and garbage the rat will be found to exist and flourish. The progress of medical knowledge within recent years has shed an informing light on the extent to which vermin such as rats, pediculi, fleas, etc., act as the carriers of organisms which produce disease. The presence of vermin indicates an inadequate hygienic standard, and they should be regarded as Nature's danger signals pointing to the existence of conditions which pave the way for disease. A rat-infected house, in the light of modern knowledge, cannot be regarded as a house fit for healthy habitation. War against the rat and the elimination of those conditions necessary to its existence must therefore be vigorously carried out as a Public Health measure.

### Malaria in England.

An exhaustive and extremely interesting report has recently been issued by the Local Government Board on the presence of indigenous malaria in England during 1917. The report contains an informing introduction by Dr. G. S. Buchanan, Assistant Medical Officer to the Board, and nine papers by various authorities dealing with the ætiology, diagnosis, prevention, and treatment of malaria contracted in this country. Interesting and important information is given regarding the presence of anopheline mosquitoes in England and the districts in which these are most frequently found. The report was issued in consequence of the occurrence in certain districts of England of cases of malaria which had evidently been contracted locally. So far as is known the total number of cases was 178, and the form of the disease was without exception "benign tertian." The disease was mild in type and no deaths occurred. As is pointed out in the introduction, the presence of indigenous malaria in England gives cause for some disquietude in view of the past history of malarial fevers in this country, and having regard to the increasing number of carriers of the malarial parasite returning to England, and to the fact that the necessary host, the anopheline mosquitoes, are commonly met with in many parts of the country. It is proposed to refer briefly in future notes to one or two of the more important questions, relating to indigenous malaria, which are discussed in the report.

### Tuberculin Treatment.

The question of the true value of tuberculin in the treatment of tuberculosis comes up again for discussion in the pages of a contemporary, and the desire is expressed for the adoption of some uniform method of administration. The practitioner who desires to try this specific form of treatment is frequently at a loss to know how to proceed owing to the variety of tuberculins at present in use, and in consequence of the different opinions held by experts as to the best method of treatment. Unfortunately in this country the methods of using tuberculin have been far too much influenced by German opinion and practice, and the failure and disappointment which followed the initial experiments with old tuberculin have been too frequently forgotten. At the outset it is necessary to realise that while a selected tuberculin will prove of value in certain well-defined forms of tuberculosis, disappointing and even disastrous results will follow if the treatment is persevered with in unsuitable cases. The best results are obtained when the treatment is commenced in a sanatorium or hospital, so that the patient may be kept under continued medical observation and supervision. By this means the beneficial effect of tuberculin in certain types of case is readily noted, while cases unsuited for this particular form of treatment are quickly recognised.

